# Release of Interleukin-1α or Interleukin-1β Depends on Mechanism of Cell Death*

**DOI:** 10.1074/jbc.M114.557561

**Published:** 2014-04-30

**Authors:** Hazel England, Holly R. Summersgill, Michelle E. Edye, Nancy J. Rothwell, David Brough

**Affiliations:** From the Faculty of Life Sciences, University of Manchester, AV Hill Building, Oxford Road, Manchester M13 9PT, United Kingdom

**Keywords:** Apoptosis, Calpain, Caspase, Cell Death, Inflammation, Interleukin, Macrophage, Necrosis (Necrotic Death)

## Abstract

The cytokine interleukin-1 (IL-1) has two main pro-inflammatory forms, IL-1α and IL-1β, which are central to host responses to infection and to damaging sterile inflammation. Processing of IL-1 precursor proteins to active cytokines commonly occurs through activation of proteases, notably caspases and calpains. These proteases are instrumental in cell death, and inflammation and cell death are closely associated, hence we sought to determine the impact of cell death pathways on IL-1 processing and release. We discovered that apoptotic regulation of caspase-8 specifically induced the processing and release of IL-1β. Conversely, necroptosis caused the processing and release of IL-1α, and this was independent of IL-1β processing and release. These data suggest that the mechanism through which an IL-1-expressing cell dies dictates the nature of the inflammatory mechanism that follows. These insights may allow modification of inflammation through the selective targeting of cell death mechanisms during disease.

## Teaser

**Background:** Cell death is a regulator of inflammation via the master cytokine IL-1.

**Results:** Apoptotic stimuli induced a caspase-8-dependent release of IL-1β from macrophages. Necroptotic stimuli induced a calpain-dependent release of IL-1α.

**Conclusion:** Mechanisms of cell death dictate the nature and form of IL-1 release.

**Significance:** Sterile inflammatory responses may be determined by the mechanisms of cell death potentially allowing for selective interventions.

## Introduction

Inflammation is one of the first and most important responses of the innate immune system to infection and injury and is known to exacerbate the pathology of major noncommunicable diseases (*e.g.* stroke, myocardial infarction, Alzheimer disease, atherosclerosis, diabetes, and cancer) (1, 2). Inflammatory cytokines associated with damaging inflammatory responses are often members of the interleukin-1 (IL-1) family, namely IL-1α and IL-1β (1). Because noncommunicable diseases kill more people than all other causes combined and are recognized as a global healthcare priority (3, 4), targeting inflammation will be central to the development of new therapeutics. Thus, understanding the signaling networks regulating the expression and cellular release of IL-1 may help to identify new therapeutic targets for the treatment of inflammatory disease.

Both IL-1α and IL-1β signal through the type 1 IL-1 receptor (IL-1R1). Stimulation of IL-1R1 causes recruitment of an accessory protein (AcP or IL-1R3), resulting in the association of the receptor complex with the adaptor molecule MyD88 causing a series of signaling steps that lead to NF-κB, p38 and JNK kinase signaling (5, 6). Prior to signaling, however, IL-1β expression must be induced in cells of the innate immune system (*e.g.* macrophages) by a danger signal because it is not expressed in healthy tissues. IL-1α is expressed constitutively in many tissues and by diverse cell types (2). Both IL-1α and IL-1β are expressed as precursors (pro-forms). Once expressed, the biologically inactive pro-IL-1β stays intracellular until a further signal activates cytosolic pattern recognition receptors, often of the NLR family, to form large multiprotein complexes called inflammasomes (7). These complexes consist of the pattern recognition receptor, pro-caspase-1, and an adaptor protein called ASC that interact via homotypic interactions between caspase activation and recruitment and pyrin domains (7). Active caspase-1 can then cleave pro-IL-1β directly to generate an active secreted molecule.

Pro-IL-1α is believed to be cleaved to a mature form by calcium-dependent proteases of the calpain family (8, 9). Pro-IL-1α is biologically active (10), but processing may increase its activity (11, 12). Although pro-IL-1α is not a substrate for caspase-1, some danger molecules can regulate an inflammasome-dependent processing and release of IL-1α (8). IL-1α can also behave like an alarmin and can be processed and released during cell death (13, 14). In this respect IL-1α has become recognized as a critical early mediator of inflammatory responses that occur after an injury or tissue necrosis (1, 2). The importance of IL-1α as a key driver of sterile inflammatory responses is now underlined by a number of clinical trials to target IL-1α in sterile diseases such as psoriasis, type 2 diabetes, and several cancers (2). Such initiatives need to be underpinned by an understanding of IL-1α processing and release mechanisms.

Recently, cell death stimuli have been proposed to regulate the processing and release of IL-1. Apoptosis has been described as a regulator of IL-1β release via mechanisms that are at least partially dependent upon caspase-8 (15–17). IL-1α, however, is suggested to be released via necrosis (13, 14). The aim of this study was to test the hypothesis that different mechanisms of cell death differentially regulated the processing and secretion of IL-1α and IL-1β.

## EXPERIMENTAL PROCEDURES

### 

#### 

##### Materials

RPMI 1640 medium and DMEM, fetal bovine serum (FBS), glutamine, and a streptomycin/penicillin antibiotic solution were all purchased from Invitrogen. Bacterial lipopolysaccharide (LPS, *Escherichia coli* 026:B6) and staurosporine (STS)^2^ were purchased from Sigma. Ac-YVAD-CHO, IETD-CHO, cycloheximide (CHX), ALLN, calpain inhibitor III, EST, and PD150606 were purchased from Merck Chemicals, Ltd. CHX causes apoptosis by inhibiting protein translation and subsequently cell growth, whereas STS is a broad spectrum kinase inhibitor that induces apoptosis. Z-VAD-fmk was purchased from Promega. Necrostatin-1 was purchased from Sigma. The anti-mouse IL-1β and IL-1α antibodies were from R&D Systems. Secondary antibody HRP conjugates were from DAKO.

##### Mice

NLRP3^−/−^ and ASC^−/−^ mice were generously provided by Dr. Vishva Dixit, Genentech (18, 19). C57BL/6J mice were purchased from Harlan UK.

##### Cell Culture

Bone marrow derived macrophages (BMDMs) were derived from the bone marrow of adult, male C57BL/6 mice as described previously (20). Cells were cultured in DMEM supplemented with 10% FCS, 100 units/ml penicillin and 100 μg/ml streptomycin, and 30% L929 supernatant containing macrophage-stimulating factor. After 6–9 days, the resulting BMDMs were plated at a density of 5 × 10^5^ cells/well and treated with LPS (1 μg/ml, 4 h) prior to incubation with apoptosis inducers (or other treatments as indicated) for 0.5–24 h as indicated.

Primary peritoneal macrophages were prepared from adult, male C57BL/6 mice, as described previously except no HEPES was added to the medium (21). Peritoneal macrophages were cultured at a density of 1 × 10^6^ cells/ml in RPMI 1640 medium supplemented with 5% FCS, 100 units/ml penicillin, and 100 μg/ml streptomycin. 24 h after culture cells were treated with LPS (1 μg/ml, 2 h) prior to a 4-h incubation with 100 μm Z-VAD (or other treatments where indicated). Supernatants and lysates were harvested for subsequent analyses. For the experiment presented in Fig. 6 the “calcium-containing” buffer was composed of (in mm): NaCl (121), KCl (5.4), MgCl_2_ (0.8), CaCl_2_ (1.8), NaHCO_3_ (6), HEPES (25), and glucose (5). The “calcium-free” buffer was composed of (in mm): NaCl (121), KCl (5.4), MgCl_2_ (0.8), NaHCO_3_ (6), HEPES (25), glucose (5), and EGTA (5).

##### Western Blotting

Supernatants and lysates were harvested and prepared in sample buffer containing 1% β-mercaptoethanol. Samples were boiled and then electrophoresed on 12% SDS-acrylamide gels. Proteins were transferred to a nitrocellulose membrane and blotted with primary antibodies, followed by HRP-conjugated secondary antibodies, and subsequent exposure using enhanced chemiluminesence reagents (ECL; Amersham Biosciences).

##### Detection of IL-1α and IL-1β by ELISA

Measurement of IL-1α and IL-1β released into macrophage culture supernatants was done using specific mouse IL-1β and IL-1α ELISA kits (R&D Systems) following the manufacturer's instructions.

##### Cell Death Assays

Cell death was recorded by measuring the release of the enzyme lactate dehydrogenase (LDH) from the cells using the CytoTox-96 assay (Promega) according to manufacturer's instructions. Cell death data are either presented as percentage LDH release compared with a Tritonlysis control or as a -fold increase over LDH release in vehicle-treated samples. Apoptosis was measured using a luminogenic Caspase-Glo 3/7 reagent according to the manufacturer's instructions (Promega).

##### Data Analysis

Data are presented as the mean ± S.D. of at least three separate cultures. Groups of data were analyzed by one-way analysis of variance followed by Bonferroni's multiple comparison test. Statistical significance was assumed when *p* < 0.05. All Western blots presented are representative of three independent experiments from three separate cultures.

## RESULTS

### 

#### 

##### Apoptotic Stimuli Induce IL-1 Release

**FIGURE 1.**
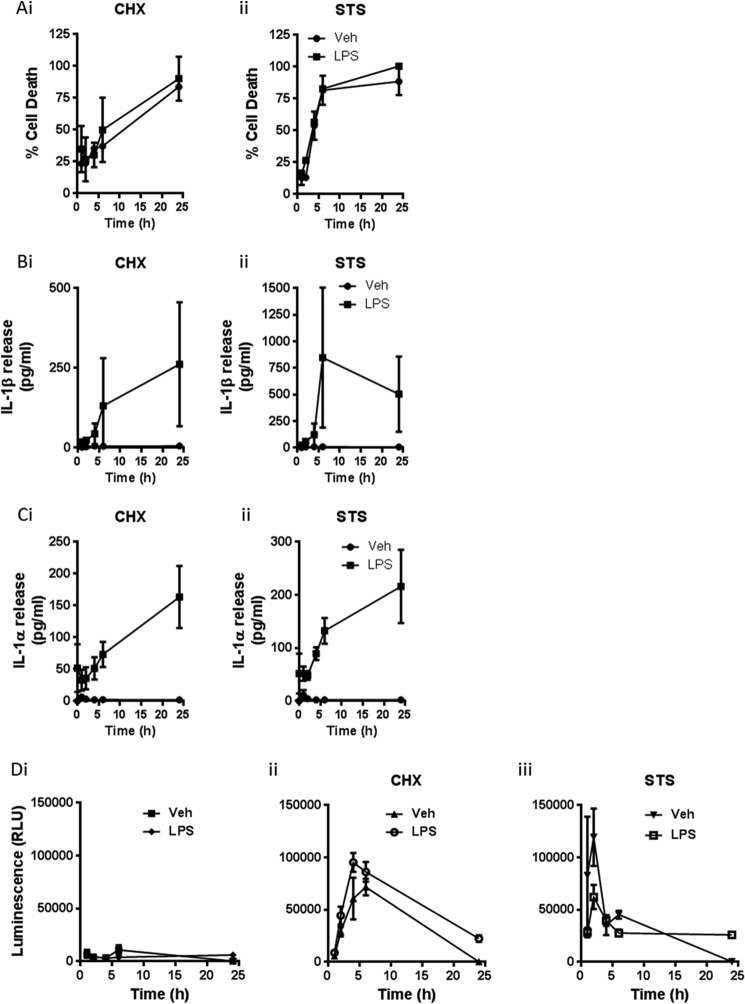
**Apoptotic stimuli induce release of IL-1.** *A–C*, mouse primary BMDMs were treated with or without LPS (1 μg/ml, 4 h) before a medium change into serum-free conditions and incubation with the apoptotic inducers CHX (100 μm) or STS (5 μm) for between 0.5 and 24 h. Supernatants were collected at the indicated times and analyzed for cell death (percentage LDH release (*A*), IL-1β release (*B*), and IL-1α release (*C*). Release of IL-1 was measured by ELISA. *D*, apoptosis was assessed by measuring activity of caspases-3 and -7 using the luminogenic Caspase-Glo 3/7 reagent. Data in *A–D* are presented as the mean ± S.D. (*error bars*) from at least three separate experiments.

BMDMs were incubated with or without LPS (1 μg/ml, 4 h) before a media change into serum-free conditions and incubation with the apoptotic inducer CHX (100 μm) or STS (5 μm), for between 0.5 and 24 h. Supernatants were collected at the indicated times and analyzed for cell death (percentage LDH release, Fig. 1*A*), IL-1β release (Fig. 1*B*), or IL-1α release (Fig. 1*C*). In a parallel experiment apoptosis was assessed using a luminogenic caspase-3/7 substrate (Fig. 1*D*). Incubation with both CHX and STS was toxic, causing an early induction of apoptosis which tapered off as the cells underwent secondary necrosis and released LDH, and this was not affected by LPS priming (Fig. 1, *A* and *D*). CHX and STS also induced the release of IL-1β (Fig. 1*B*) and IL-1α (Fig. 1*C*). However, this occurred only in LPS-primed cells. Thus, using two classic inducers of apoptosis we were able to stimulate the release of both IL-1β and IL-1α from LPS-primed BMDMs.

##### Apoptosis-induced IL-1β Release Is Caspase-8-dependent

**FIGURE 2.**
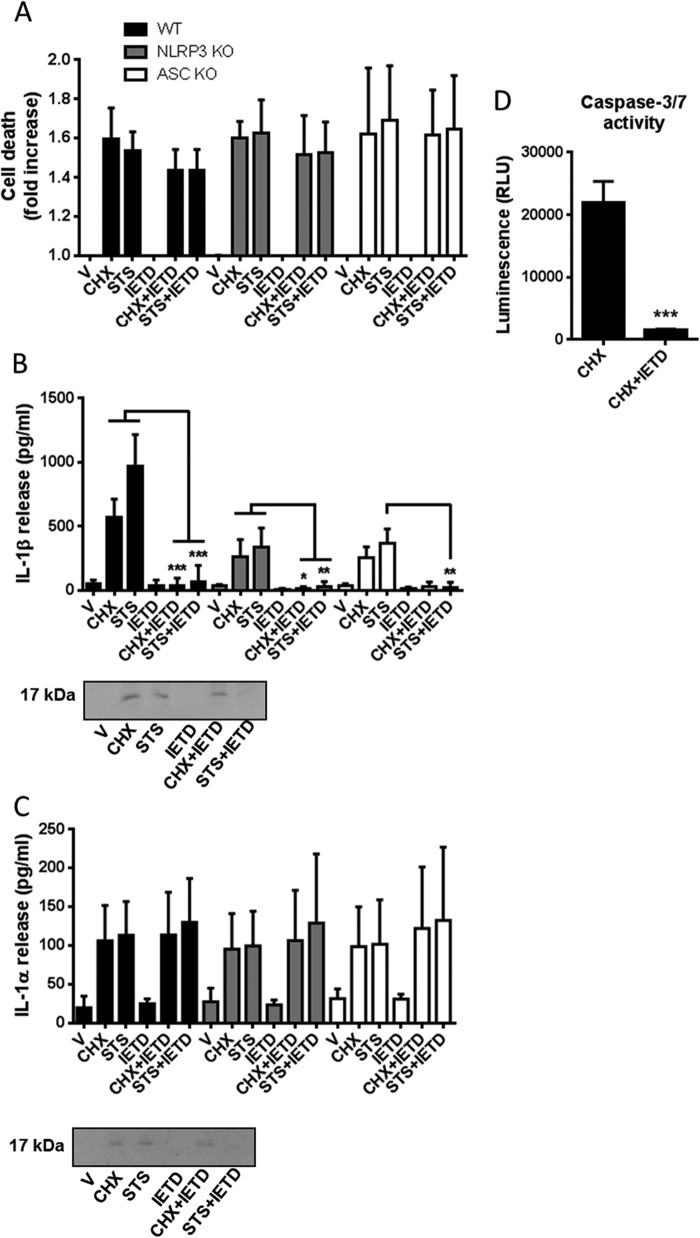
**Apoptotic stimuli induce the processing and release of IL-1β and IL-1α via distinct mechanisms.** BMDMs isolated from WT, NLRP3^−/−^, or ASC^−/−^ mice were treated with LPS (1 μg/ml, 4 h) and then treated with CHX (100 μm) or STS (5 μm) in serum-free medium for 24 h. Cells were also incubated with or without the caspase-8 inhibitor IETD (100 μm) where indicated for the duration of the experiment (after LPS stimulation). *A–C*, supernatants were collected and analyzed for cell death (-fold increase LDH release over vehicle treatment (*A*), IL-1β release (*B*), and IL-1α release (*C*). Release of IL-1 was measured by ELISA. *D*, the effects of IETD against apoptosis induced by CHX were measured using the luminogenic Caspase-Glo 3/7 reagent. Identification of the mature 17-kDa IL-1 species in supernatants was confirmed by Western blotting. All data are presented as the mean ± S.D. (*error bars*) from at least three separate experiments. Blots shown are representative. ***, *p* < 0.001; **, *p* < 0.01.

Apoptosis has been reported to activate the NLRP3 inflammasome/caspase-1 to induce IL-1β release (15) and also by inflammasome-independent but caspase-8-dependent mechanisms (16, 17). Thus, we sought to determine the mechanisms regulating CHX- and STS-induced IL-1β and IL-1α release from LPS-primed BMDMs. BMDMs isolated from WT, NLRP3^−/−^, or ASC^−/−^ mice were treated with LPS (1 μg/ml, 4 h) and then treated with CHX (100 μm) or STS (5 μm) in serum-free medium for 24 h because we had established that at this time robust death and IL-1 release were observed (Fig. 1). Cell death in response to CHX or STS was unaffected in NLRP3^−/−^ or ASC^−/−^ compared with WT (Fig. 2*A*). Furthermore, cell death measured by LDH release in response to CHX or STS was also unaffected by incubation with the selective caspase-8 inhibitor IETD (Fig. 2*A*). CHX- or STS-induced IL-1β processing and release were partially inhibited by the loss of NLRP3 (*p* < 0.001) or ASC (*p* < 0.01 (CHX), *p* < 0.001 (STS)) compared with WT and were completely inhibited by IETD (Fig. 2*B*). Importantly, IETD also inhibited apoptosis induced by CHX measured using caspase-3/7 activation (Fig. 2*D*). CHX and STS induced significant IL-1α processing and release (*p* < 0.01) compared with vehicle treatment, and this was not affected by inflammasome knockout or by caspase-8 inhibition (Fig. 2*C*). Thus, these data suggest that apoptotic stimuli induced a caspase-8-dependent apoptosis and regulation of IL-1β processing and release, with a partial involvement of the NLRP3 inflammasome, whereas release of IL-1α followed necrotic cell death.

**FIGURE 3.**
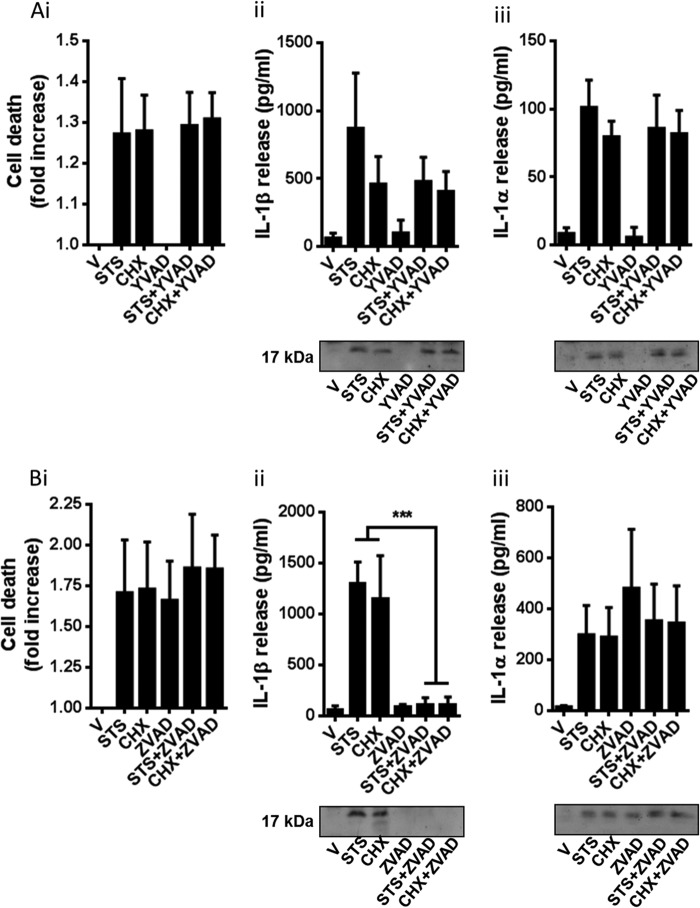
**Effects of YVAD and Z-VAD on apoptotic stimuli induced IL-1 release.** BMDMs isolated from WT mice were treated with LPS (1 μg/ml, 4 h) and then treated with CHX (100 μm) or STS (5 μm) in serum-free medium for 24 h. Cells were also incubated with or without the caspase-1 inhibitor YVAD (100 μm (*A*) or the pan-caspase inhibitor Z-VAD (100 μm, *B*), where indicated for the duration of the experiment (after LPS stimulation). Supernatants were collected and analyzed for cell death (-fold increase in LDH release over vehicle treatment (*i*), IL-1β release (*ii*), and IL-1α release (*iii*). Release of IL-1 was measured by ELISA. Identification of the mature 17-kDa IL-1 species in supernatants was confirmed by Western blotting. All data are presented as the mean ± S.D. (*error bars*) from at least three separate experiments. Blots shown are representative. ***, *p* < 0.001.

Our data above suggested only a partial role for caspase-1 in the processing and release of IL-1β in response to CHX and STS. This was confirmed using the caspase-1 inhibitor YVAD (100 μm) which barely affected STS- or CHX-induced IL-1β release and had no effect on cell death or IL-1α release, in LPS-primed BMDMs (Fig. 3*A*). Incubation of LPS-primed BMDMs with the pan-caspase inhibitor Z-VAD (100 μm) completely inhibited STS- and CHX-induced IL-1β processing and release, but had no effect on STS- or CHX-induced cell death or IL-1α release (Fig. 3*B*). Whereas the caspase-8 inhibitor IETD and the caspase-1 inhibitor YVAD had no effect on cell death alone when incubated with LPS-primed BMDMs (Figs. 2*A* and 3*A*), Z-VAD induced cell death and significant IL-1α processing and release (Fig. 3*B*).

##### Necroptosis Induces IL-1α Release

**FIGURE 4.**
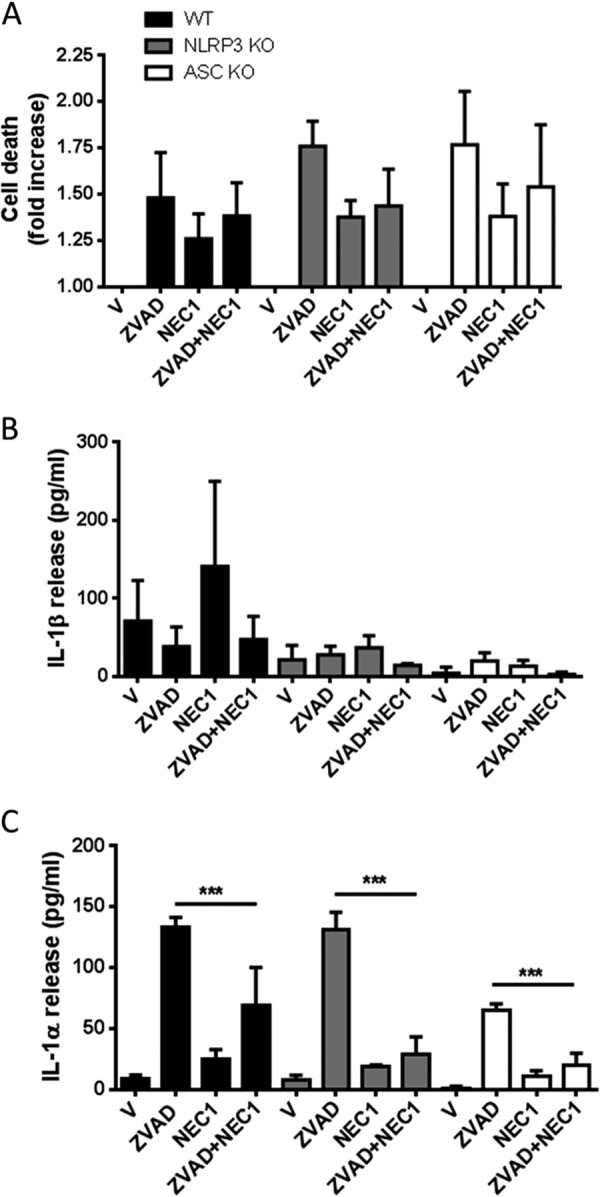
**Effects of necrostatin-1 on LPS- and Z-VAD-induced cell death and IL-1 release in BMDMs.** BMDMs isolated from WT, NLRP3^−/−^, or ASC^−/−^ mice were treated with LPS (1 μg/ml, 4 h) and then treated with vehicle (0.5% dimethyl sulfoxide) or Z-VAD (100 μm) in serum-free medium for 20 h. Cells were also incubated with or without the RIP1 inhibitor NEC1 (100 μm) where indicated for the duration of the experiment (after LPS stimulation). Supernatants were collected and analyzed for cell death (-fold increase in LDH release over vehicle treatment (*A*), IL-1β release (*B*), and IL-1α release (*C*). Release of IL-1 was measured by ELISA. All data are presented as the mean ± S.D. (*error bars*) from at least three separate experiments. ***, *p* < 0.001.

**FIGURE 5.**
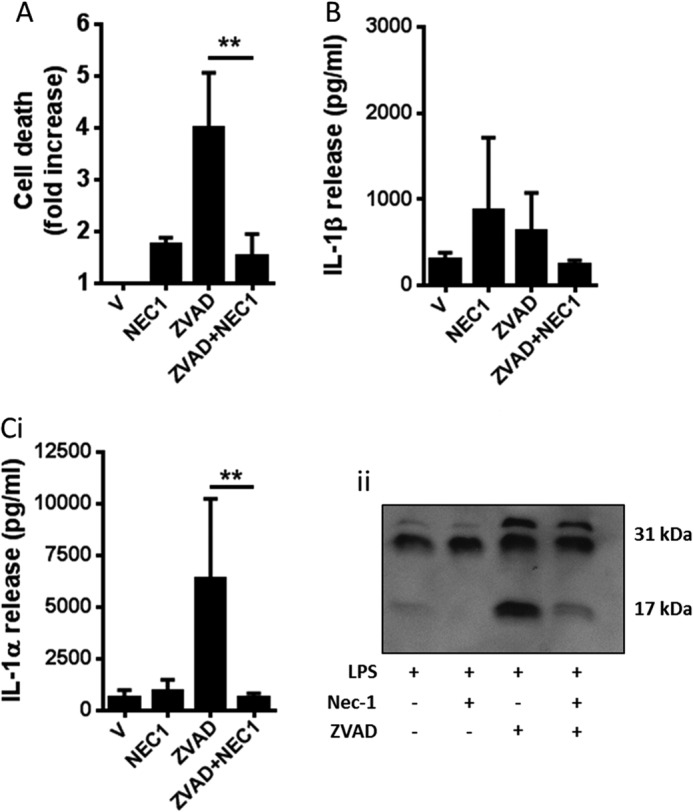
**Effects of necrostatin-1 on LPS- and Z-VAD-induced cell death and IL-1 release in peritoneal macrophages.** Mouse primary peritoneal macrophages were primed with LPS (1 μg/ml, 2 h), followed by 4-h treatment with vehicle (0.5% dimethyl sulfoxide) or Z-VAD (100 μm). Cells were also incubated with or without the RIP1 inhibitor NEC1 (100 μm) where indicated for the duration of the experiment (after LPS stimulation). Supernatants were collected and analyzed for cell death -fold increase in LDH release over vehicle treatment (*A*), IL-1β release (*B*), and IL-1α release (*C*, *i* and *ii*). Release of IL-1 was measured by ELISA. Identification of the mature 17-kDa IL-1 species in supernatants was confirmed by Western blotting. All data are presented as the mean ± S.D. (*error bars*) from at least three separate experiments. Blots shown are representative. **, *p* < 0.01.

Treatment of macrophages with LPS and the pan-caspase inhibitor Z-VAD induces RIP3-dependent necroptosis (22), a caspase-independent form of programmed necrosis. Necroptosis can be inhibited by the RIP1 inhibitor necrostatin-1 (NEC1) (23, 24). Thus, the data we present above (Fig. 3*B*) suggested that in our LPS-primed BMDMs, Z-VAD induced necroptosis, that this drove IL-1α processing and release, and that this was completely independent of IL-1β. To further confirm that necroptosis was regulating the processing and release of IL-1α we incubated LPS-primed BMDMs with Z-VAD (100 μm) in the absence or presence of the RIP1 inhibitor NEC1 (100 μm). To determine whether necroptotic responses to LPS and Z-VAD were inflammasome-dependent we also repeated the LPS and Z-VAD experiment in BMDMs isolated from NLRP3^−/−^ and ASC^−/−^ mice (Fig. 4). Z-VAD induced cell death in LPS-primed BMDMs from WT, NLRP3^−/−^, and ASC^−/−^ mice, and this was not significantly affected by NEC1 (Fig. 4*A*). In LPS-primed BMDMs from all strains Z-VAD induced IL-1α release, and this was significantly inhibited by NEC1 (Fig. 4*C*). There was no release of IL-1β over vehicle treatment (Fig. 4*B*). Interestingly, the release of IL-1α from LPS- and Z-VAD-treated ASC^−/−^ cells was lower than from WT or NLRP3^−/−^ cells, suggesting that under these conditions release of IL-1α may be partially ASC-dependent (Fig. 4*C*). We repeated these experiments in primary mouse peritoneal macrophages, another cell type commonly used to interrogate mechanisms of IL-1 release. In LPS-primed primary peritoneal macrophages, NEC1 (100 μm) inhibited Z-VAD-induced cell death (100 μm, 4-h treatment) (Fig. 5*A*) and processing and release of IL-1α (Fig. 5*C*). Again, under these conditions, release of IL-1β did not occur (Fig. 5*B*). These data suggest that necroptosis regulates the release of IL-1α independently of the inflammasome and IL-1β.

##### Necroptosis-induced IL-1α Release Is Ca^2+^- and Calpain-dependent

**FIGURE 6.**
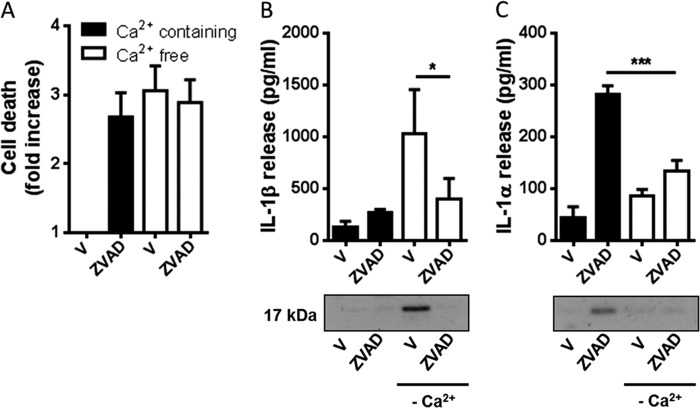
**Necroptosis-induced IL-1α release is Ca^2+^-dependent.** BMDMs isolated from WT mice were treated with LPS (1 μg/ml, 4 h) and then treated with vehicle (0.5% dimethyl sulfoxide) or Z-VAD (100 μm) in Ca^2+^-containing or Ca^2+^-free medium for 24 h. Supernatants were collected and analyzed for cell death (-fold increase in LDH release over vehicle treatment (*A*), IL-1β release (*B*), and IL-1α release (*C*). Release of IL-1 was measured by ELISA. Identification of the mature 17-kDa IL-1 species in supernatants was confirmed by Western blotting. All data are presented as the mean ± S.D. (*error bars*) from at least three separate experiments. Blots shown are representative. ***, *p* < 0.001; *, *p* < 0.05.

Pro-IL-1α is believed to be cleaved by calcium (Ca^2+^)-dependent proteases of the calpain family (8, 9). To determine the nature of IL-1α processing during necroptosis we first stimulated LPS-primed BMDMs with Z-VAD (100 μm) in Ca^2+^-containing or Ca^2+^-free medium (Fig. 6). Incubation of the cells in Ca^2+^-free medium (24 h) alone induced cell death comparable with Z-VAD treatment (Fig. 6*A*) and was also a sufficient trigger for IL-1β processing and release (Fig. 6*B*). LPS-primed cells incubated in Ca^2+^-free conditions in the presence of Z-VAD did not secrete IL-1β (Fig. 6*B*). In contrast, Z-VAD induced IL-1α processing and release in Ca^2+^-containing medium, and this was inhibited in Ca^2+^-free conditions (Fig. 6*C*). These data suggest that the processing and release of IL-1α during necroptosis are Ca^2+^-dependent.

**FIGURE 7.**
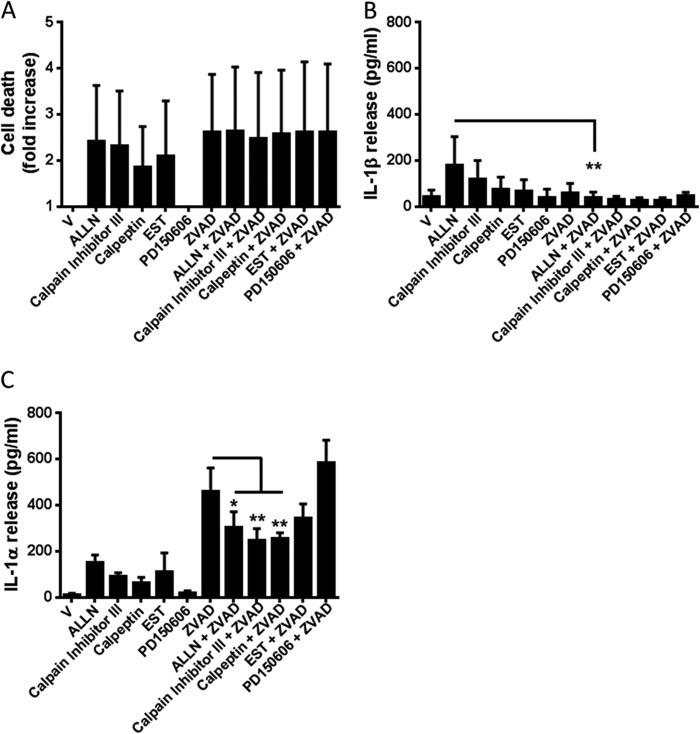
**Necroptosis-induced IL-1α release is calpain-dependent.** BMDMs isolated from WT mice were treated with LPS (1 μg/ml, 4 h) and then treated with vehicle (0.5% dimethyl sulfoxide) or Z-VAD (100 μm). Cells were also co-incubated with the following calpain I inhibitors: ALLN (100 μm), calpain inhibitor III (100 μm), calpeptin (100 μm), EST (100 μm), or PD150606 (50 μm) for 24 h. Supernatants were collected and analyzed for cell death (-fold increase in LDH release over vehicle treatment (*A*), IL-1β release (*B*), and IL-1α release (*C*). Release of IL-1 was measured by ELISA. All data are presented as the mean ± S.D. (*error bars*) from at least three separate experiments. **, *p* < 0.01; *, *p* < 0.05.

An anti-calpain I antibody blocked Ca^2+^-dependent processing of pro-IL-1α in lysates U937 histiocytic lymphoma cells (25). We therefore tested a panel of calpain I inhibitors against the effects of Z-VAD in LPS-primed BMDMs (Fig. 7). Over the course of this experiment several of the calpain inhibitors caused cell death, and Z-VAD-induced cell death was not inhibited by calpain inhibition (Fig. 7*A*). Related to its effects on cell death the calpain inhibitor ALLN (100 μm) induced some IL-1β release (Fig. 7*B*). This was not related to necroptosis however because it was inhibited by Z-VAD (Fig. 7*B*). Furthermore, none of the calpain inhibitors tested induced levels of IL-1α release comparable with Z-VAD (Fig. 7*C*). ALLN (100 μm), calpain inhibitor III (MDL-28170, 100 μm), and calpeptin (100 μm) significantly inhibited Z-VAD-induced IL-1α release (Fig. 7*C*). Together these data suggest that necroptosis drives the processing and release of IL-1α through a mechanism that is Ca^2+^-dependent and at least partially dependent upon calpains.

## DISCUSSION

We report here that the inflammatory mechanisms triggered by dying cells depend upon the nature of cell death. In LPS-primed BMDMs, apoptosis inducing stimuli drove a caspase-8-dependent processing and release of IL-1β (Fig. 2). Release of mature IL-1α also occurred, but this was independent of caspase-8. Thus the mechanisms regulating the release of these two cytokines were dissociated under these conditions. IL-1α release may result simply from a loss of membrane integrity during necrosis (13, 14). This may be the case here in response to the apoptosis inducers CHX or STS where its release correlated closely with that of LDH, a marker of cell viability. Our data on apoptosis induced IL-1β release are entirely consistent with a recent report showing that STS, and another apoptotic agent, doxorubicin, induced a caspase-8-dependent processing and release of IL-1β from LPS-primed dendritic cells (17). As in our study, the caspase-1 inhibitor YVAD was minimally effective in reducing apoptosis-induced IL-1β release (17). This recent work demonstrated that in LPS-primed dendritic cells caspase-8-dependent IL-1β release is minimally dependent upon the NLRP3 inflammasome (17). In experiments reported here in LPS-primed BMDMs lacking the inflammasome components, NLRP3 or ASC, STS- or CHX-induced IL-1β release was significantly reduced, whereas cell death and IL-1α release were unaffected (Fig. 2). The caspase-8 inhibitor IETD completely abolished IL-1β release, without affecting IL-1α release (Fig. 2). Hence under these conditions, there is a partial inflammasome-dependent regulation of caspase-8. This is entirely consistent with recent literature where *Salmonella* infection of macrophages reportedly caused recruitment of caspase-8 to an ASC inflammasome (26). It is also consistent with a recent article reporting that caspase-8 co-localizes with caspase-1 in LPS- and ATP-treated macrophages and that caspase-8 activation in response to LPS and ATP requires components of the NLRP3 inflammasome (27). Our data add to these recent reports by suggesting that caspase-8 is a key IL-1-processing enzyme. The processing and release of IL-1α in response to certain DAMPs (damage-associated molecular patterns) are suggested to be NLRP3-inflammasome dependent (8), but under the experimental conditions used here, the processing and secretion of IL-1α appeared to be completely independent of the pathway regulating processing and secretion of IL-1β.

Cell death has been traditionally viewed as apoptotic (programmed) or necrotic (uncontrolled). However, the view that necrotic cell death is a rapid and uncontrolled loss of membrane integrity has been challenged by a growing body of literature describing signaling mechanisms orchestrating necrotic cell death, which has led to a new definition of necroptosis or programmed necrosis (28). Necroptosis is a caspase-independent cell death that is typically induced by the activation of death receptors such as FAS and TNF, although caspase inhibitors in some cellular systems also induce necroptotic processes (29). There are no biochemical or morphological features that reveal the mechanism through which an apparently necrotic cell has died. However, necroptosis is known to require the formation of a pro-necroptotic complex containing the kinases RIP1 and RIP3 (30, 31) and is inhibited by the RIP1 inhibitor NEC1 (23, 24). Our observation above, that during necroptosis there was no processing or release of IL-1β, suggests that the processing and release of IL-1β are processes that depend upon caspase activity. Processing and release of IL-1α were independent of caspases and were inhibited by the RIP1 inhibitor NEC1 (Figs. 4 and 5). Necroptotic cell death in response to LPS and Z-VAD proceeded normally in BMDMs isolated from NLRP3^−/−^ and ASC^−/−^ mice, suggesting that necroptosis occurs independently of the inflammasome (Fig. 4).

NEC1 is protective in sterile injury models such as ischemia/reperfusion injury in the heart (32) and the brain (23, 33), paradigms of injury in which IL-1 blockade is also protective (34, 35). RIP1-dependent production of IL-1α has been linked to inflammatory tissue damage in a genetic mouse model of inflammation, although in this instance the production of IL-1α was independent of RIP3 and necroptotic cell death (36). Thus, it is possible that necroptosis contributes to cell death-induced IL-1α processing and release during sterile inflammatory disease. We have reported recently that IL-1α-dependent inflammation worsens outcome in a rat model of subarachnoid hemorrhage, a devastating brain injury for which there are limited clinical options (37). Within this study we reported that heme, a product of red blood cell lysis, induces the release of IL-1α from LPS-primed cultures of mixed glia, without inducing the release of IL-1β (37). Heme-induced cell death in astrocytes (38) and macrophages (39) is inhibited by NEC1. Thus, it is possible that necroptosis could be a major regulator of IL-1α-dependent inflammation *in vivo*.

Elucidating the interaction between cell death and the processing and release of IL-1 family cytokines is central to understanding mechanisms of sterile inflammation. In this study we have reported that the pathways regulating the processing and release of the pro-inflammatory cytokines IL-1α and IL-1β occur independently of each other and that necroptosis is a mechanism that regulates processing and release of IL-1α independently of IL-1β. These insights identify the possibility that inflammation occurring during injury or disease could be inhibited by targeting specific mechanisms of cell death in specific cell types.

## Footnotes

2 The abbreviations used are:

STS: staurosporine
BMDM: bone marrow-derived macrophage
CHX: cycloheximide
fmk: fluoromethyl ketone
LDH: lactate dehydrogenase
NEC1: necrostatin-1
Z: benyloxycarbonyl
ALLN: *N*-acetyl-Leu-Leu-Nle-al
EST: epoxysuccinyl-l-leucylamido-3-methylbutane ethyl ester
RIP: receptor-interacting protein kinase.
